# Higher risk, lower recurrence: A year of risk-adapted twist-drill craniostomy with pressure-controlled fibrinolytic irrigation therapy for chronic subdural hematoma

**DOI:** 10.1016/j.bas.2026.107487

**Published:** 2026-10-21

**Authors:** Roberto Doria-Medina, Marco Bissolo, István Csók, Lucas Becker, Manou Overstijns, Mazin Omer, Mukesch Johannes Shah, Jürgen Beck, Roland Roelz

**Affiliations:** a Department of Neurosurgery, Medical Center – University of Freiburg, Faculty of Medicine, University of Freiburg, Germany; b Department of Neurosurgery, Ruhr-University Bochum, Universitaetsklinikum Knappschaftskrankenhaus Bochum GmbH, Bochum, Germany; c Department of Neuroradiology, Medical Center – University of Freiburg, Faculty of Medicine, University of Freiburg, Germany

**Keywords:** Chronic subdural hematoma, Fibrinolysis, Recurrence, Subdural irrigation, Twist-drill craniostomy, Urokinase

## Abstract

**Introduction:**

Recurrence remains challenging in chronic subdural hematoma (cSDH). Twist-drill craniostomy (TDC) permits bedside treatment, but incomplete evacuation limits its effectiveness in membranous hematomas. Thus, we introduced TDC with pressure-controlled fibrinolytic irrigation therapy (TDC-FIT).

**Research question:**

Within an institutional risk-adapted treatment algorithm, how does the reoperation risk compare after TDC-FIT versus TDC?

**Material and methods:**

All patients undergoing cSDH surgery in 2025 were included. TDC-FIT was preferentially used for recurrent and/or membranous cSDH. The primary outcome was ipsilateral recurrent/persistent cSDH requiring surgery <3 months. Procedure-level analyses used robust patient-clustered standard errors, and patient-level sensitivity analyses.

**Results:**

208 procedures were performed in 139 patients; 191 procedures in 130 patients were eligible for the primary analysis. Reoperation occurred after 2/39 TDC-FIT (5.1%) and 35/152 TDC procedures (23.0%; RR 0.22, 95% CI 0.05–0.93; P = 0.040), an absolute risk difference of −17.9 percentage points. The adjusted RR was 0.16 (95% CI 0.03–0.74; P = 0.020). Patient-level RRs were 0.25 for index surgery, 0.15 for first recurrent or persistent episode, and 0.17 for membranous configurations; estimates were imprecise. Procedure-related complications occurred after 6/43 TDC-FIT procedures and 12/165 TDC procedures (RR 1.92, 95% CI 0.75–4.91; P = 0.174).

**Conclusion and discussion:**

Within this risk-adapted algorithm, TDC-FIT was associated with fewer reoperations, despite preferential use in higher-risk cSDH. Patient-level estimates were directionally consistent but imprecise. Complications were numerically more frequent after TDC-FIT, and equivalent safety cannot be inferred. Randomized studies are needed to establish effectiveness and safety.

## Highlights

• TDC-FIT was preferentially used for higher-risk hematomas.
• TDC-FIT had 5.1% reoperation versus 23.0% with TDC.
• Patient-level estimates favored TDC-FIT but were imprecise.
• Both modalities show a similar complication profile.

## 1 Introduction

Chronic subdural hematoma (cSDH) is common and disproportionately affects older and medically vulnerable patients (Balser et al., 2015; Uno et al., 2017; Rauhala et al., 2019; Tommiska et al., 2022; Dziho et al., 2025). Symptomatic cSDH generally requires timely treatment, if safely possible, because surgical delay may worsen functional outcome (Knuutinen et al., 2025). At the same time, frailty and comorbidity increase perioperative risk, making approaches that limit surgical and anesthetic burden particularly attractive (Stubbs et al., 2020; Sastry et al., 2021). Twist-drill craniostomy (TDC) with closed subdural drainage can be performed at the bedside under local anesthesia, offering a resource-sparing alternative to larger operations with minimal medical and surgical trauma (e.g. avoiding general anesthesia, skin depressions from burr holes, independent of operating room scheduling, etc.) (Smely et al., 1997; Reinges et al., 1998; Singh et al., 2011; Yagnik et al., 2021). Although TDC is minimally invasive and resource-sparing, concerns about recurrence have limited its broader adoption (Singh et al., 2011; Yagnik et al., 2021). Open craniotomy with membranectomy has therefore been used for selected refractory cases, especially in organized or recurrent hematomas (Rocchi et al., 2007; Van Der et al., 2014; Matsumoto et al., 2018), while middle meningeal artery (MMA) embolization has emerged as a surgical adjunct or alternative strategy (Fiorella et al., 2025; Liu et al., 2024; Davies et al., 2024; Shankar et al., 2026; Shotar et al., 2025; Kellner et al., 2026), even though recent randomized trials have reported heterogeneous efficacy estimates across populations, embolic agents, and outcome definitions.

Unsatisfied with our recurrence rate, particularly in membranous hematoma configurations, we introduced TDC with pressure-controlled fibrinolytic irrigation therapy (TDC-FIT) (Doria-Medina et al., 2025; Bissolo et al., 2026) to reduce reoperation rates while preserving a minimally invasive bedside approach, minimize postoperative morbidity, and reduce adjunctive therapy such as MMA embolization. Based on earlier institutional experience and observed recurrence patterns, we implemented a risk-adapted algorithm that preferentially assigned TDC-FIT to membranous and recurrent or persistent cSDH (Doria-Medina et al., 2025; Bissolo et al., 2026). The present study describes the first full year of its structured implementation, examines associations with three-month reoperation rates at the procedural and patient level, and records procedure-related complications and functional outcome. The primary research question concerns ipsilateral reoperation after TDC-FIT versus conventional TDC within this risk-adapted treatment approach.

## 2 Materials and methods

### 2.1 Study design and cohort

We conducted a single-center observational implementation study at a tertiary referral center, using prospectively collected clinical data that were analyzed retrospectively. All consecutive patients undergoing surgical treatment for cSDH between January 1 and December 31, 2025, were included.

At the patient level, characteristics and functional outcomes were recorded once per patient, using the earliest treated presentation as baseline. At the procedure level, each treated side was considered separately. Patients could contribute multiple procedures and could receive both treatment modalities. Repeated and bilateral procedures were linked to the corresponding patient for clustered analyses.

The overall cohort comprised 208 procedures performed in 139 patients. The primary recurrence analysis was restricted to 191 procedures in 130 patients with a technically completed initial procedure and an assessable recurrence outcome. This included persistent hematomas requiring further evacuation despite correct initial drain placement. All 208 procedures were included in the safety analysis.

Reporting was guided by the Strengthening the Reporting of Observational Studies in Epidemiology (STROBE) recommendations for cohort studies. The STROBE checklist is provided in the supplementary material.

### 2.2 Risk-adapted treatment allocation and procedures

Based on our previous institutional findings, recurrent or persistent cSDH and membranous morphology were considered higher-risk configurations for recurrence requiring surgery (Doria-Medina et al., 2025, Doria-Medina et al., 2026; Bissolo et al., 2026). TDC-FIT was preferentially used for these configurations when clinically and technically feasible (Fig. 1). Conventional TDC was generally used for first-presentation cSDH without membranous morphology and when TDC-FIT was unavailable or unsuitable (e.g. inability to cooperate safely with the irrigation procedure).

**Fig. 1:**
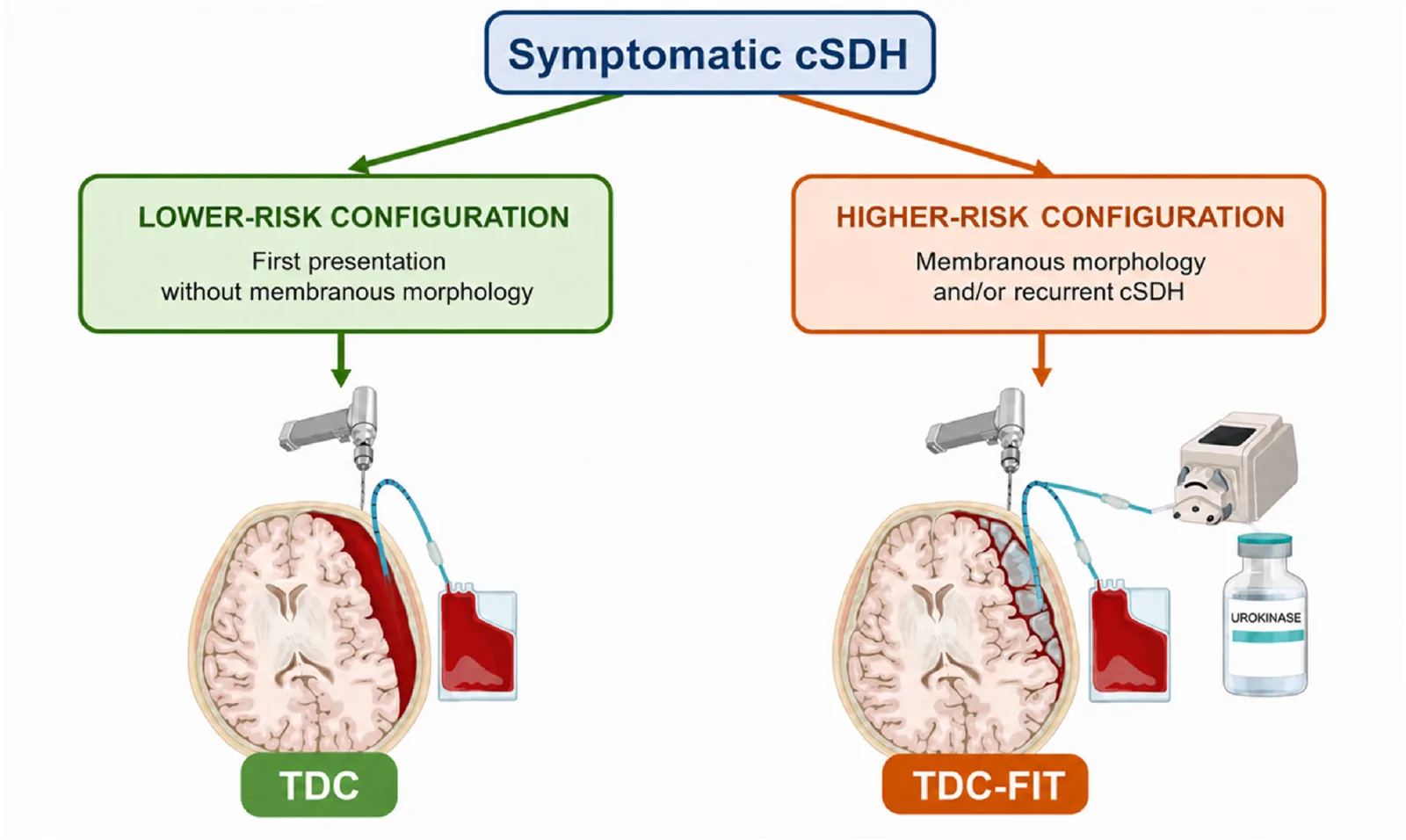
Institutional risk-adapted treatment algorithm for the use of TDC-FIT Abbreviations: cSDH, chronic subdural hematoma; TDC, twist-drill craniostomy; TDC-FIT, twist-drill craniostomy with pressure-controlled fibrinolytic irrigation therapy. The figure demonstrates the concept of our institutional risk-adapted treatment algorithm for the use of twist-drill craniostomy with pressure-controlled fibrinolytic irrigation therapy in chronic subdural hematoma considered higher-risk for recurrence. In cases of symptomatic chronic subdural hematoma, our standard of care was to perform a conventional twist-drill craniostomy and placement of a passive subdural drain (left). In case of membranous hematoma configuration, based on preoperative imaging assessment, or in case of recurrent or persistent hematoma, pressure-controlled fibrinolytic irrigation therapy was conducted via a two-lumen subdural drain (right).

Conventional TDC was performed at the bedside under local anesthesia at the frontal or parietal eminence, followed by placement of a passive subdural drain. The drain was generally removed after 2–3 days when drainage had ceased. TDC-FIT used a similar twist-drill approach with a double-lumen silicone catheter connected to the LiquoGuard® 7 CSF Management System (Möller Medical GmbH, Germany) for the pressure-controlled irrigation with urokinase solution. Irrigation was usually continued for 3–5 days until hematoma clearance, followed by passive drainage until outflow ceased and subsequent catheter removal. Thus, irrigation duration was guided by clearance and drainage rather than fixed treatment interval. Further technical details have been described previously (Doria-Medina et al., 2025; Bissolo et al., 2025a, 2025b, 2026).

### 2.3 Outcome definitions

The primary outcome was ipsilateral recurrent or persistent cSDH requiring reoperation within three months after an eligible treated-side procedure. In simultaneous bilateral procedures, each treated side was evaluated separately in the primary procedure-level analysis. Repeat interventions included conventional TDC, burr-hole craniostomy, craniotomy, or TDC-FIT. Surgery for cSDH was indicated by neurological symptoms, clinical deterioration, or progressive mass effect on follow-up imaging. Persistent cSDH included an insufficiently evacuated hematoma requiring an additional ipsilateral procedure despite a technically completed initial operation with correct subdural drain placement.

Procedures were excluded from the primary recurrence analysis when technical failure prevented effective subdural drainage or when the three-month recurrence outcome could not be assessed, as detailed in Supplementary Table S1. A qualifying reoperation occurring before death remained an outcome event. Excluded procedures were retained in the full-cohort description and safety analysis. In the conservative full-cohort sensitivity analysis, all 17 primary-excluded procedures were classified as treatment failures, together with qualifying reoperations after primary-included procedures. This broader composite endpoint was interpreted separately from recurrence.

Three patient-level sensitivity analyses were performed: (1) the earliest eligible episode with membranous morphology, whether a first presentation or a recurrent or persistent episode; (2) each patient's index surgical episode; and (3) the first eligible recurrent or persistent episode. Each patient contributed only one episode per analysis. Simultaneous bilateral procedures were combined, and reoperation on either treated side constituted an event; for unilateral episodes, only ipsilateral reoperation counted. Patients could contribute to more than one sensitivity analysis. The analyses were therefore interpreted separately and were not pooled.

Safety was assessed in all 208 procedures. Procedure-related complications were defined as adverse events directly attributable to the operation. Distinct complications following separate procedures in the same patient were counted separately. Complications were classified according to Landriel-Ibañez (Landriel Ibañez et al., 2011). Grade I events managed without invasive treatment were classified as minor. Grades II–IV, involving invasive treatment, intensive care management for life-threatening complications, or death, were classified as major. Systemic medical complications without direct surgical attribution were recorded separately at the patient level and were not included in the procedure-related safety endpoint.

Functional outcome was assessed using the modified Rankin Scale (mRS). The preoperative mRS from the earliest treated presentation was compared with the latest documented postoperative assessment. The mRS was summarized in two categories, mRS 0–3 and mRS 4–6. Deaths were coded as mRS 6. Follow-up duration was measured from the patient's earliest treated presentation to the latest documented follow-up date. All-cause mortality within three months was classified as occurring during hospitalization or after discharge.

### 2.4 Clinical and radiological variables

Age, sex, laterality, antithrombotic therapy, membranes presence, procedure type, time from radiological diagnosis to surgery, mRS, complications, mortality clinical and radiological follow-up, and adjunctive MMA embolization were recorded. Hematoma morphology was classified according to the Nakaguchi classification (Nakaguchi et al., 2001); laminar and trabecular types were considered membranous cSDH. At the patient level, membranous morphology was considered present if identified on either side of a for simultaneous bilateral presentations.

Antithrombotic medication was categorized as none, antiplatelet therapy, vitamin K antagonist (VKA), direct oral anticoagulant (DOAC), and dual antithrombotic therapy (any combination of two antithrombotic agents). In case a patient was taking any antithrombotic medication, our institutional practice was as follows: (1) the medication was immediately discontinued, (2) VKA was reversed with prothrombin complex concentrate (PCC) and vitamin K1, (3) DOAC was managed with 3500 IU PCC, followed by 1000 IU PCC every 12 h for 48 h, and (4) surgery was only performed after coagulation parameters and platelet counts met institutional thresholds. Specifically, surgery was performed once the following institutional thresholds were met: (1) platelet count of at least 70 × 10^9^/L, (2) international normalized ratio below 1.2, and (3) partial thromboplastin time < 38 s. In most anticoagulated patients, this allowed surgery within 24 h of hematoma presentation.

### 2.5 Statistical analysis

Categorical variables are reported as counts and percentages, and continuous variables as medians with interquartile ranges (IQR). The ordinal mRS was summarized as categories 0–3 and 4–6. Procedure-level characteristics were summarized descriptively without conventional significance testing because treatment allocation was non-random and risk-adapted, and patients could contribute multiple procedures.

The primary analysis concerned ipsilateral reoperation after each eligible treated-side procedure. Bilateral and repeat procedures represented distinct treatment exposures, but procedures contributed by the same patient were not assumed to be independent. Risk ratios (RRs) were estimated using modified Poisson regression with a log link, and the absolute risk differences using linear probability models. All procedure-level models used patient-clustered robust standard errors with a finite-sample correction (CR1) and normal reference Wald inference for two-sided P values and 95% confidence intervals (CIs). The RR remained the principal relative effect measure with absolute risk difference reported as complimentary measure.

An exploratory multivariable modified Poisson model estimated adjusted RRs after adjustment for membranous morphology and recurrent or persistent presentation, using the same patient-clustered covariance method. Adjusted estimates were considered exploratory because of the sparse TDC-FIT outcome events and limited treatment overlap.

The full-cohort treatment-failure sensitivity analysis used the same patient-clustered modified Poisson and linear probability models. For patient-level sensitivity analyses, RRs were reported with log-Wald 95% CIs, and comparisons used two-sided Fisher exact tests. No adjusted patient-level models were fitted because of the small sample sizes. No adjustment for multiple comparisons was applied, and the sensitivity analyses were interpreted as exploratory.

Procedure-related complication RRs were estimated using modified Poisson regression with patient-clustered robust standard errors and the same finite-sample correction. Complication subtypes, patient-level complication burden, and severe systemic medical events were summarized descriptively. Distinct complications following different procedures in the same patient were counted separately at the procedure level. For the categorical mRS comparison, paired preoperative and postoperative categories (0–3 versus 4–6) were compared using a two-sided exact McNemar test.

All analyses were exploratory and two-sided, with P < 0.05 considered statistically significant. Analyses were performed using R version 4.6.1 in RStudio (Posit Software, version 2026.08.1 + 195).

### 2.6 Ethical considerations

The study adhered to ethical guidelines and was conducted in accordance with the Declaration of Helsinki. Institutional Review Board approval was obtained before the initiation of the study (number 24-1201-S1-retro and 24-1157-S1). Patient confidentiality and data anonymization were ensured throughout the research process. To ensure the complete inclusion of all treated patients in this retrospective study, written consent was waived by the Institutional Review Board.

## 3 Results

### 3.1 Overall patient and procedural cohorts

During 2025, 208 procedures were performed in 139 patients, comprising 43 TDC-FIT procedures (20.7%) and 165 conventional TDC procedures (79.3%). Fifty-two patients (37.4%) underwent more than one procedure. Fourteen patients (10.1%) received TDC-FIT only, 101 (72.7%) received conventional TDC only, and 24 (17.3%) received both treatments.

Median patient age was 78 years (IQR, 69.5–85.0), and 92 patients (66.2%) were male. Membranous hematoma was identified in 74/138 patients (53.6%), and 50 patients (36.0%) were receiving antithrombotic therapy. Information on hematoma configuration was missing in one patient. Six patients (4.3%) underwent MMA embolization. Reoperation within three months occurred in 31/131 patients (23.7%). Eight patients had no assessable recurrence outcome. Patient-level characteristics and outcomes of the overall cohort are summarized in Table 1, Panels A and B.

**Table 1:** Patient-level characteristics and outcomes of the overall cohort.

A. Patient-level characteristics	Overall cohort (N = 139)
Procedures, n	208
Patients with > 1 procedure	52 (37.4)
Patients with TDC-FIT only	14 (10.1)
Patients with conventional TDC only	101 (72.7)
Patients treated with both modalities	24 (17.3)
Age, years, median (IQR)	78 (69.5–85.0)
Sex	
Male	92 (66.2)
Female	47 (33.8)
Laterality	
Right	63 (45.3)
Left	61 (43.9)
Bilateral	15 (10.8)
Membranous hematoma^a^	74 (53.6)
Any antithrombotic therapy	50 (36.0)
Antithrombotic therapy	
None	89 (64.0)
Antiplatelet therapy	26 (18.7)
VKA	7 (5.0)
DOAC	12 (8.6)
Dual antithrombotic therapy	5 (3.6)
Preoperative mRS 0–3	69 (49.6)
Preoperative mRS 4–6	70 (50.4)
MMA embolization	6 (4.3)
**B. Patient-level outcomes**	
Ipsilateral reoperation within 3 months^b^	31 (23.7)
Clinical and radiological follow-up, days, median (IQR)	122 (78–189)
Follow-up to latest assessment among survivors, days, median (IQR)	123 (83–189)
Latest postoperative mRS	
mRS 0–3	123 (88.5)
mRS 4–6	16 (11.5)
Postoperative mRS, median (IQR)	0 (0–2)
Improved postoperative mRS	116 (83.5)
Unchanged postoperative mRS	12 (8.6)
Worsened postoperative mRS	11 (7.9)
All-cause mortality within 3 months	9 (6.5)
During hospitalization	3 (2.2)
After discharge	6 (4.3)

Data are presented as n (%) unless otherwise indicated. Patients were counted once, except for the procedure count. Baseline characteristics refer to the earliest treated presentation. Simultaneous bilateral procedures were combined, with membranes considered present if seen on either side. mRS categories were available for all 139 patients; death was coded as mRS 6.

Abbreviations: DOAC, direct oral anticoagulant; MMA, middle meningeal artery; mRS, modified Rankin Scale; TDC, twist-drill craniostomy; TDC-FIT, twist-drill craniostomy with pressure-controlled fibrinolytic irrigation therapy; VKA, vitamin K antagonist.

a Membrane data: n = 138.

b Eight of the patients who died before the 3-month assessment had no assessable reoperation outcome. These patients were excluded, thus, the overall reoperation rate was calculated on a cohort of 131 patients.

Seventeen of the 208 procedures (four TDC-FIT and 13 conventional TDC) were excluded from the primary recurrence analysis: three because of premature drain removal or dislocation, three because o drain placement in an unintended intracranial compartment, six because of acute postoperative hemorrhage requiring urgent surgery, three because of in-hospital death, and two because of death after discharge within three months. Each procedure was assigned one principal exclusion reason. The primary analysis therefore included 191 procedures in 130 patients with an assessable ipsilateral recurrent or persistent cSDH outcome (Supplementary Table S1).

### 3.2 Primary procedure-level analysis

The primary analysis included 39 TDC-FIT procedures (20.4%) and 152 conventional TDC procedures (79.6%). TDC-FIT was preferentially used for higher-risk configurations, whereas conventional TDC was preferentially used for first presentations. Membranous morphology was present in 35/39 TDC-FIT procedures (89.7%) and 68/152 conventional TDC procedures (44.7%). Recurrent or persistent presentations accounted for 22/39 TDC-FIT procedures (56.4%) and 14/152 conventional TDC procedures (9.2%). No TDC-FIT procedure occurred in the non-membranous first-presentation stratum. Procedure-level characteristics are presented in Table 2. Of 191 procedures, 153 (79.6%) were performed within 24 h, 29 (15.2%) at 24–48 h, 7 (3.7%) at 48–72 h, and 3 (1.6%) at ≥72 h.

**Table 2:** Characteristics of the procedure cohort.

	Overall (N = 191)	TDC-FIT (n = 39)	TDC (n = 152)
Patients^a^	130	36	116
Age, years, median (IQR)	79 (70–85)	82 (70.5–85)	78 (69.5–85)
Sex			
Male	135 (70.7)	29 (74.4)	106 (69.7)
Female	56 (29.3)	10 (25.6)	46 (30.3)
Treated side			
Right	94 (49.2)	21 (53.8)	73 (48.0)
Left	97 (50.8)	18 (46.2)	79 (52.0)
Membranous morphology	103 (53.9)	35 (89.7)	68 (44.4)
First-presentation procedure	155 (81.2)	17 (43.6)	138 (90.8)
Recurrent or persistent procedure	36 (18.8)	22 (56.4)	14 (9.2)
Any antithrombotic therapy	62 (32.5)	8 (20.5)	54 (35.5)
None	129 (67.5)	31 (79.5)	98 (64.5)
Antiplatelet therapy	35 (18.3)	5 (12.8)	30 (19.7)
VKA	9 (4.7)	1 (2.6)	8 (5.3)
DOAC	12 (6.3)	1 (2.6)	11 (7.2)
Dual antithrombotic therapy	6 (3.1)	1 (2.6)	5 (3.3)
Preoperative mRS (0–3)	100 (52.4)	23 (59.0)	77 (50.7)
Preoperative mRS (4–6)	91 (47.6)	16 (41.0)	75 (49.3)
Diagnosis-to-surgery interval, days, median (IQR) [range]	0 (0–0) [0–9]	0 (0–0) [0–5]	0 (0–0) [0–9]

Data are n (%) unless otherwise indicated and presented per procedure, except for patient counts. No conventional P values are provided for baseline characteristics because patients could contribute repeated procedures. Twenty-two patients received both treatments within this analysis cohort.

Abbreviations: DOAC, direct oral anticoagulant; mRS, modified Rankin Scale; TDC, twist-drill craniostomy; TDC-FIT, twist-drill craniostomy with pressure-controlled fibrinolytic irrigation therapy; VKA, vitamin K antagonist.

a The treatment-specific patient counts overlap because 22 patients contributed procedures to both treatment groups.

Reoperation occurred after 2/39 TDC-FIT procedures (5.1%) and 35/152 conventional TDC procedures (23.0%). In the patient-clustered analysis, TDC-FIT was associated with a lower risk of reoperation (RR 0.22, 95% CI 0.05–0.93; P = 0.040). The absolute risk difference was −17.9 percentage points (95% CI −28.5 to −7.3; P = 0.001; Table 3, Panel A).

**Table 3:** Primary and sensitivity effectiveness analyses.

Outcome or comparison	TDC-FIT n/N (%)	Conventional TDC n/N (%)	Estimate (95% CI)	P value
**A. Primary procedure-level analysis (191 procedures; 130 patients)**				
Reoperation	2/39 (5.1)	35/152 (23.0)	—	—
Risk difference, percentage points	—	—	−17.9 (−28.5 to −7.3)	0.001
RR	—	—	0.22 (0.05–0.93)	0.040
**B. Adjusted patient-clustered model: adjusted RR (191 procedures; 130 patients)**				
TDC-FIT versus conventional TDC	—	—	0.16 (0.03–0.74)	0.020
Membranous versus non-membranous	—	—	2.11 (1.11–4.02)	0.023
Recurrent/persistent versus first presentation	—	—	1.17 (0.57–2.37)	0.670
**C. Full-cohort treatment-failure analysis (208 procedures; 139 patients)**				
Treatment failure	6/43 (14.0)	48/165 (29.1)	—	—
Risk difference, percentage points	—	—	−15.1 (−28.2 to −2.1)	0.023
RR	—	—	0.48 (0.22–1.05)	0.066
**D. Patient-level sensitivity analyses: reoperation and unadjusted RR**				
Earliest eligible membranous episode	1/19 (5.3)	17/55 (30.9)	0.17 (0.02–1.19)	0.030
Index surgical episode	1/16 (6.3)	28/114 (24.6)	0.25 (0.04–1.74)	0.120
First eligible recurrent/persistent episode	1/18 (5.6)	4/11 (36.4)	0.15 (0.02–1.20)	0.054

Panels A–C: RRs were estimated using modified Poisson regression and absolute risk differences using linear probability models, with patient-clustered CR1 covariance and normal-reference Wald inference. RR was the principal relative effect measure; P values are model-specific. Panel B includes treatment, membranous morphology, and recurrent/persistent presentation in one model; estimates are exploratory because only two TDC-FIT events occurred and treatment overlap was limited. Panel C counts all 17 primary-excluded procedures as treatment failures in addition to the 37 primary reoperations; this is a broader endpoint than recurrence.

Panel D: each patient contributed one episode per analysis; bilateral procedures were combined and reoperation on either treated side counted. For unilateral episodes, only ipsilateral procedures counted. RR CIs use the log-Wald method; P values use two-sided Fisher exact tests. Because these methods differ, a Fisher P value below 0.05 can coexist with an RR C1 including 1. Patients could contribute to more than one of the three analyses; these were not pooled or adjusted for multiple testing.

Abbreviations: CI, confidence interval; RR, risk ratio; TDC, twist-drill craniostomy; TDC-FIT, twist-drill craniostomy with pressure-controlled fibrinolytic irrigation therapy.

After adjustment for membranous morphology and recurrent or persistent presentation, the adjusted RR was 0.16 (95% CI 0.03–0.74; P = 0.020). These estimates were considered exploratory because sparse events and limited treatment overlap (Table 3, Panel B).

### 3.3 Sensitivity analyses

In the full-cohort sensitivity analysis of 208 procedures in 139 patients, treatment failure occurred after 6/43 TDC-FIT procedures (14.0%) and 48/165 conventional TDC procedures (29.1%). These totals comprised the two and 35 primary reoperations plus the four and 13 primary-excluded procedures, respectively. The patient-clustered RR was 0.48 (95% CI 0.22–1.05; P = 0.066) and the absolute risk difference was −15.1 percentage points (95% CI –28.2 to −2.1; P = 0.023). The direction of the association was consistent with the primary analysis, but the principal RR comparison did not reach statistical significance (Table 3, Panel C).

Among 74 patients with membranous morphology, reoperation occurred in 1/19 patients treated with TDC-FIT (5.3%) and 17/55 treated with conventional TDC (30.9%; RR 0.17, 95% CI 0.02–1.19; Fisher exact P = 0.030). The index-episode analysis included 130 patients with assessable outcomes: reoperation occurred in 1/16 TDC-FIT patients (6.3%) and in 28/114 conventional TDC patients (24.6%; RR 0.26, 95% CI 0.04–1.74; Fisher exact P = 0.120). Among 29 patients contributing a first eligible recurrent or persistent episode, reoperation occurred in 1/18 treated with TDC-FIT (5.6%) and in 4/11 treated with conventional TDC (36.4%; RR 0.15 95% CI 0.02–1.20; Fisher exact P = 0.054). All three estimates favored TDC-FIT but were imprecise (Table 3, Panel D).

### 3.4 Safety outcomes

Procedure-related complications occurred after 6/43 TDC-FIT procedures (14.0%) and 12/165 conventional TDC procedures (7.3%; patient-clustered RR 1.92, 95% CI 0.75–4.91; P = 0.174). Major complications occurred after 5/43 TDC-FIT procedures (11.6%) and 11/165 conventional TDC-procedures (6.7%; RR 1.74, 95% CI 0.63–4.85; P = 0.286). Minor complications occurred after 1/43 TDC-FIT procedure (2.3%) and 1/165 conventional TDC procedure (0.6%; RR 3.84, 95% CI 0.24–60.98; P = 0.341). The 18 complication-bearing procedures involved 17 patients. Complication rates were numerically higher after TDC-FIT, the differences were not statistically significant, but the wide CIs did not exclude clinically important harm.

At the patient level, 17/139 patients (12.2%), experiences at least one procedure-related complication, including 15 patients (10.8%) with a major complication and two (1.4%) with a minor complication. One patient experienced two distinct postoperative hemorrhages after two separate conventional TDC procedures, counted as two procedure-level events but as one affected patient. In addition, four patients (2.9%) treated with conventional TDC experienced severe systemic medical events; none were recorded after TDC-FIT. These events were summarized separately from procedure-related complications and were not compared statistically (Table 4, Panel B).

**Table 4:** Procedure- and patient-level complications in the full safety cohort.

	TDC-FIT (n = 43)	TDC (n = 165)	Patient-clustered RR (95% CI)	P value
**A. Procedure-level safety outcomes**				
Any complication	6 (14.0)	12 (7.3)	1.92 (0.75–4.91)	0.174
Major complications	5 (11.6)	11 (6.7)	1.74 (0.63–4.85)	0.286
Drain dislocation requiring invasive treatment	2 (4.7)	3 (1.8)		
Catheter placement in unintended intracranial compartment	2 (4.7)	2 (1.2)		
Postoperative hemorrhage requiring urgent evacuation	1 (2.3)	5 (3.0)		
Subdural empyema	0 (0.0)	1 (0.6)		
Minor complications	1 (2.3)	1 (0.6)	3.84 (0.24–60.98)	0.341
Drain dislocation not requiring invasive treatment	1 (2.3)	1 (0.6)		
**B. Patient-level safety outcome (N = 139)**		**Overall cohort**		
≥ 1 procedure-related complication		17 (12.2)		
≥ 1 major procedure-related complication		15 (10.8)		
≥ 1 minor procedure-related complication		2 (1.4)		
≥ 1 severe systemic medical event		4 (2.9)		

Data are presented as n (%) unless otherwise indicated. Major complications required invasive treatment (grade II), intensive care for a life-threatening event (grade III), or resulted in death (grade IV). Minor complications were managed without invasive treatment and caused no serious sequelae (grade I). Distinct events after separate procedures in the same patient were counted separately. RRs and P values were obtained using modified Poisson regression with robust standard errors clustered by patient. Individual complication subtypes and patient-level outcomes were analyzed descriptively. Patient-level rows are not mutually exclusive because a patient could experience both a procedure-related and systemic event.

Abbreviations: TDC, twist-drill craniostomy; TDC-FIT, twist-drill craniostomy with pressure-controlled fibrinolytic irrigation therapy.

### 3.5 Functional outcome, follow-up, and mortality

Preoperative and postoperative mRS categories were available for all 139 patients, including nine deaths coded as mRS 6. The proportion with mRS 0–3 increased from 69/139 (49.6%) preoperatively to 123/139 (88.5) at the latest documented assessment; mRS 4–6 occurred in 70/139 (50.4%) and 16/139 (11.5%), respectively. Fifty-seven patients changed from mRS 4–6 to 0–3; three changed in the opposite direction, and 79 remained in the same category (exact McNemar P < 0.001). Median clinical and radiological follow-up was 122 days (IQR 78–189). Nine patients (6.5%) died within three months, three (2.2%) during hospitalization and six (4.3%) after discharge (Table 1, Panel B)

## 4 Discussion

This study describes outcomes following the selective use of TDC-FIT within a risk-adapted treatment approach for cSDH. TDC-FIT was associated with fewer ipsilateral reoperations within three months than conventional TDC (5.1% versus 23.0%; RR 0.22; absolute risk difference −17.9 percentage points). Earlier institutional cohorts reported procedural-level reoperation rates of approximately 30% after conventional TDC, although follow-up periods and cohort definitions differed (Doria-Medina et al., 2025, Doria-Medina et al., 2026; Bissolo et al., 2026). This is notable because TDC-FIT was preferentially used for membranous and recurrent cSDH, configurations expected to carry a higher risk of incomplete evacuation and reoperation (Singh et al., 2011; Doria-Medina et al., 2025, Doria-Medina et al., 2026; Bissolo et al., 2026; Jack et al., 2015; Motiei-Langroudi et al., 2018). In fact, in the patient-level analysis on membranous hematoma presentation, reoperation rates were 5.3% for TDC-FIT, while conventional TDC presented a 30.9% reoperation rate, an absolute observed difference of −25.6 percentage points. The full-cohort treatment-failure RR and most patient-level comparisons were not statistically significant. Their direction was consistent with the primary result, but the wide intervals leave considerable uncertainty; non-significance should not be attributed to sample size alone. However, the membranous patient-level analysis supports further investigation in this subgroup.

The higher event rates in the full-cohort analysis do not represent additional recurrences detected during follow-up. Its treatment-failure endpoint deliberately counted all 17 primary-excluded procedures as failures, increasing events from 2 to 6 after TDC-FIT and from 35 to 48 after conventional TDC. It therefore combines the recurrence with technical failure and otherwise unevaluable procedures. The attenuated RR and CI show that the apparent treatment advantage was less certain under this broader definition. This analysis complements, but cannot validate or replace, the recurrence-specific analysis.

Current pathophysiological understanding of cSDH describes it as an inflammatory and angiogenic process driven by neomembrane formation, fragile vasculature, and repeated microhemorrhage (Edlmann et al., 2017; Holl et al., 2018; De Simone et al., 2026). Complement-related inflammatory activity has also been identified in cSDH and may contribute to hematoma persistence (Marklund et al., 2025). In our previous studies, membrane presence emerged as one of the strongest predictors of reoperation (Doria-Medina et al., 2025; Doria-Medina et al., 2026; Bissolo et al., 2026). Pressure-controlled irrigation and local fibrinolysis may improve catheter drainage of consolidated components, thereby addressing a mechanical limitation of conventional TDC (Doria-Medina et al., 2025; Bissolo et al., 2025a, 2025b, 2026). However, the present study did not measure hematoma volume, septation, residual volume, membrane resolution, or biological hematoma markers. The proposed mechanism therefore remains speculative.

TDC-FIT was deliberately introduced for configurations considered at higher risk of recurrence (Fig. 1) (Doria-Medina et al., 2026). Accordingly, membranes were more frequent in the TDC-FIT group (89.7% versus 44.7%), and more than half of TDC-FIT procedures treated recurrent or persistent hematomas. The observed association is compatible with improved evacuation in these configurations, but non-random treatment allocation prevents a causal conclusion. Some membranous hematomas were treated with conventional TDC. Device availability and patient suitability may have influenced this selection, but the reasons were not recorded systematically.

Our use of TDC reflects an established bedside pathway under local anesthesia, with limited surgical exposure and reduced dependence on operating-room availability (Knuutinen et al., 2025; Stubbs et al., 2020; Sastry et al., 2021; Smely et al., 1997; Reinges et al., 1998; Singh et al., 2011; Yagnik et al., 2021). This have also led to a near non-existing use of open craniotomies for consolidated cSDH as demonstrated in an earlier study (Bissolo et al., 2026). In 2025, we performed no burr-hole or open craniotomies for cSDH. These practical considerations may be relevant for older or medically vulnerable patients, but do not establish superiority over burr-hole drainage, which can also be performed under local anesthesia. Recurrence after conventional TDC is an important limitation, particularly in organized or membranous hematomas (Singh et al., 2011; Yagnik et al., 2021). The 23.0% conventional-TDC reoperation rate in this cohort may have increased the observed absolute difference and limits generalizability to centers using other drainage techniques. The control-arm event rates in MAGIC-MT and EMBOLISE were approximately 10%, but their populations, surgical strategies, and endpoints differed from ours (Liu et al., 2024; Davies et al., 2024). Cross-trial comparisons therefore cannot establish wheter TDC-FIT is preferable to burr-hole drainage.

Procedure-related complications were numerically more frequent after TDC-FIT than conventional TDC (14.0% versus 7.3%; RR1.92). Although the difference was not statistically significant, the 95% CI of 0.75–4.91 was compatible with a clinically important increase in complications. Most events involved catheter displacement or placement error. The small number of TDC-FIT procedures and adverse events prevents a reliable assessment of relative safety; these data do not demonstrate equivalence or non-inferiority. Larger prospective studies should evaluate recurrence and complications together.

Functional outcome was overall favorable in both groups with 83.5% of patients improved. The proportion of patients with mRS 0–3 increased from 49.5% preoperatively to 88.5% at the latest assessment. Nine patients died within three months, including three during hospitalization and six after discharge. Although mortality was present only in patients treated with conventional TDC, this should not be attributed to either procedure in this non-randomized cohort. Future studies would benefit from structured patient-reported outcome, length of stay, readmission rates, treatment burden, and cost.

MMA embolization has gained attention as an adjunctive treatment for cSDH. Six patients (4.3%) in this series underwent MMA embolization, two in the TDC-FIT group and four in the conventional TDC group. Achieving recurrence rates around 5% with TDC-FIT could potentially reduce the need for adjunctive MMA embolization, which in itself carries additional procedural risks and costs (Fiorella et al., 2025; Liu et al., 2024; Davies et al., 2024; Shankar et al., 2026; Shotar et al., 2025; Debs et al., 2025). However, this study was not designed to compare TDC-FIT with MMA embolization or to evaluate whether irrigation reduces need for embolization; no conclusion can be drawn regarding their relative efficacy. TDC-FIT aims to improve hematoma evacuation, whereas MMA embolization targets the vascular supply to the neomembrane; these mechanisms may be complementary. The limited use of MMA embolization reflects our local practice as a consequence of the increased use of TDC-FIT and does not establish that TDC-FIT reduces the need for embolization.

## 5 Limitations

This single-center observational study reflects the early implementation of an institutional treatment algorithm and was analyzed retrospectively. Treatment allocation was intentionally not randomized and strongly related to membranous morphology and recurrent or persistent presentation. No TDC-FIT procedure occurred in the non-membranous first-presentation stratum, indicating limited overlap between groups. Propensity-score matching could restrict comparison to patients with overlapping treatment probabilities, but would discard observation and change the target population in this small cohort. It would not address unmeasured confounding. We therefore retained the patient-clustered analyses and explicitly consider adjusted estimates exploratory, particularly given only two TDC-FIT reoperations. Important radiological and clinical determinants of treatment selection were not recorded systematically, and statistical adjustment cannot remove this uncertainty. The endpoint captured recurrence requiring surgery, not all radiographic recurrence. The small treatment and event counts limit precision for recurrence and safety. Patient clustering accounts for correlated observations but not for time-varying confounding or the influence of earlier treatment outcomes on subsequent treatment selection. The patient-level sensitivity analyses avoid repeated observations within each analysis but remain small, overlap with one another, and were not adjusted for multiple testing. Excluding technically unsuccessful or otherwise unevaluable procedures may introduce selection bias. The full-cohort composite retains these procedures but evaluates a different endpoint and does not eliminate this bias.

## 6 Conclusions

During the first year of a risk-adapted institutional algorithm, TDC-FIT was associated with fewer ipsilateral reoperations than conventional TDC, despite its preferential use for higher-risk recurrent and membranous cSDH. Patient-level estimates were directional consistent but imprecise. Procedure-related complications were more frequent after TDC-FIT, but differences did not reach significance. TDC-FIT might offer a promising surgical alternative for higher-risk cSDH configurations, but prospective controlled studies are required to confirm its efficacy and safety and to define its role relative to conventional TDC or other surgical approaches, or adjunctive MMA embolization.

## Author contributions

Roberto Doria-Medina and Roland Roelz conceptualized and designed the study. Roberto Doria-Medina, Marco Bissolo, István Csók, Lucas Becker, Manou Overstijns, and Mazin Omer collected the data. Roberto Doria-Medina and Marco Bissolo conducted the investigation, data curation, and project administration. Roberto Doria-Medina performed the analysis and interpreted the data. Roberto Doria-Medina, Marco Bissolo and Roland Roelz drafted the manuscript, with critical revisions from Jürgen Beck, and Mukesch Johannes Shah. All authors reviewed and approved the final manuscript.

## Disclosures

The authors report no personal, financial, or institutional relationships related to the materials or devices described in this manuscript that could be construed as a conflict of interest.

## Declaration of generative AI and AI-assisted technologies in the manuscript preparation process

During preparation of this work, the authors used AI tools to improve language and readability. After using this service, the authors reviewed and edited the content as needed and take full responsibility for the content of the published article.

## Declaration of competing interest

The authors declare that they have no known competing financial interests or personal relationships that could have appeared to influence the work reported in this paper.
