# Supplementary material for: Higher risk, lower recurrence: A year of risk-adapted twist-drill craniostomy with pressure-controlled fibrinolytic irrigation therapy for chronic subdural hematoma
Source: Brain Spine. 2026 Oct 21;6:107487. doi: 10.1016/j.bas.2026.107487 (PMC13718344; doi:10.1016/j.bas.2026.107487)
Supplement: Supplementary file 1 [file mmc1.docx]

| **Supplementary Table S1.** Reasons for exclusion from the primary recurrence analysis. | | |
| --- | --- | --- |
| **Primary reason for exclusion** | **TDC-FIT** | **TDC** |
| Premature drain removal/dislocation | 2 | 1 |
| Drain placement in unintended intracranial compartment | 1 | 2 |
| Acute rebleeding requiring urgent surgery | 1 | 5 |
| In-hospital mortality | 0 | 3 |
| Death after discharge, within three months | 0 | 2 |
| **Total** | **4** | **13** |

Each excluded procedure was counted once. Technical complications or hemorrhage took precedence over a later death for the reporting allocation; the death rows therefore do not represent all deaths in the cohort.

Abbreviations: TDC, twist-drill craniostomy; TDC-FIT, twist-drill craniostomy with pressure-controlled fibrinolytic irrigation therapy
