# Supplementary material for: Higher risk, lower recurrence: A year of risk-adapted twist-drill craniostomy with pressure-controlled fibrinolytic irrigation therapy for chronic subdural hematoma
Source: Brain Spine. 2026 Oct 21;6:107487. doi: 10.1016/j.bas.2026.107487 (PMC13718344; doi:10.1016/j.bas.2026.107487)
Supplement: Supplementary file 2 [file mmc2.docx]

STROBE Statement—checklist of items that should be included in reports of observational studies

|  | Item No. | Recommendation | Page  No. | Relevant text from manuscript |
| --- | --- | --- | --- | --- |
| **Title and abstract** | 1 | (*a*) Indicate the study’s design with a commonly used term in the title or the abstract | Abstract Methods and Section 2.1 |  |
|  |  | (*b*) Provide in the abstract an informative and balanced summary of what was done and what was found | Abstract |  |
| Introduction | | | |  |
| Background/rationale | 2 | Explain the scientific background and rationale for the investigation being reported | Introduction | “Based on earlier institutional experience and observed recurrence patterns, we implemented a risk-adapted algorithm that preferentially assigned TDC-FIT to membranous and recurrent or persistent cSDH”. |
| Objectives | 3 | State specific objectives, including any prespecified hypotheses | End of Introduction. | Compare ipsilateral reoperation within three months after TDC-FIT versus conventional TDC in a risk-adapted treatment approach. |
| Methods | | | |  |
| Study design | 4 | Present key elements of study design early in the paper | Opening of Section 2.1. | Single-centre observational cohort; treatment was nonrandom and selected clinically. |
| Setting | 5 | Describe the setting, locations, and relevant dates, including periods of recruitment, exposure, follow-up, and data collection | Section 2.1 | The recruitment period was the calendar year 2025 with three months follow-up |
| Participants | 6 | (*a*) *Cohort study*—Give the eligibility criteria, and the sources and methods of selection of participants. Describe methods of follow-up  *Case-control study*—Give the eligibility criteria, and the sources and methods of case ascertainment and control selection. Give the rationale for the choice of cases and controls  *Cross-sectional study*—Give the eligibility criteria, and the sources and methods of selection of participants | Sections 2.1 and 2.3; Supplementary Table S1. | Overall cohort: 208 procedures in 139 patients. Primary cohort: 191 procedures in 130 patients after 17 procedure exclusions. |
|  |  | (*b*) *Cohort study*—For matched studies, give matching criteria and number of exposed and unexposed  *Case-control study*—For matched studies, give matching criteria and the number of controls per case | N/A | N/A |
| Variables | 7 | Clearly define all outcomes, exposures, predictors, potential confounders, and effect modifiers. Give diagnostic criteria, if applicable | Sections 2.2 to 2.4. | Exposure is TDC-FIT or conventional TDC. The primary endpoint is ipsilateral recurrent or persistent cSDH requiring reoperation within three months after an eligible treated-side procedure. Technical failure, complication-related surgery and the full-cohort composite are distinguished. |
| Data sources/ measurement | 8* | For each variable of interest, give sources of data and details of methods of assessment (measurement). Describe comparability of assessment methods if there is more than one group | Sections 2.3 and 2.4. |  |
| Bias | 9 | Describe any efforts to address potential sources of bias | Section 2.5 and Limitations. | Within-patient dependence is handled in variance estimates; adjustment includes morphology and recurrent/persistent presentation. Patient-level and full-cohort sensitivity analyses examine alternative populations/endpoints. These measures do not remove confounding by indication or post-treatment selection. |
| Study size | 10 | Explain how the study size was arrived at | Section 2.1. | The study includes the available consecutive cohort during the defined period. |

Continued on next page

| Quantitative variables | 11 | Explain how quantitative variables were handled in the analyses. If applicable, describe which groupings were chosen and why | Section 2.5 + Tables | Continuous variables are summarized as medians and IQRs; categorical variables as counts and percentages. Models use treatment, binary morphology and binary recurrent/persistent presentation |
| --- | --- | --- | --- | --- |
| Statistical methods | 12 | (*a*) Describe all statistical methods, including those used to control for confounding | Section 2.5 | Linear probability, modified Poisson and logistic models estimate RD, RR and OR with patient-clustered CR1 covariance and normal-reference Wald inference. Adjusted models include morphology and recurrent/persistent presentation. |
|  |  | (*b*) Describe any methods used to examine subgroups and interactions | Sections 2.3 and 2.5. | Separate patient-level analyses select the true index episode, earliest eligible membranous episode and first eligible recurrent/persistent episode. They are exploratory. |
|  |  | (*c*) Explain how missing data were addressed | Section 2.5; Tables | Missing values were not imputed; denominators reflect available outcomes/covariates. |
|  |  | (*d*) *Cohort study*—If applicable, explain how loss to follow-up was addressed  *Case-control study*—If applicable, explain how matching of cases and controls was addressed  *Cross-sectional study*—If applicable, describe analytical methods taking account of sampling strategy | Section 2.3 and Tables | Unevaluable recurrence outcomes were not coded as absence of recurrence. Explained how death, complications preventing assessment and lost follow-up were addressed. The composite sensitivity is not an imputation of recurrence. |
|  |  | (*e*) Describe any sensitivity analyses | Sections 2.3 and 2.5 |  |
| Results | | | | |
| Participants | 13* | (a) Report numbers of individuals at each stage of study—eg numbers potentially eligible, examined for eligibility, confirmed eligible, included in the study, completing follow-up, and analysed | Sections 3.1 to 3.3; Tables 1 to 3. | Overall: 208 procedures/139 patients; primary: 191/130. Patient sensitivities: index 130, membranous 74, recurrent/persistent 29. |
|  |  | (b) Give reasons for non-participation at each stage | Supplementary Table S1. | Seventeen exclusions: displacement 3, wrong compartment 3, hemorrhage 6, in-hospital death 3, post-discharge death 2. Four were TDC-FIT and 13 TDC. |
|  |  | (c) Consider use of a flow diagram | Figure 1 | Institutional treatment-algorithm |
| Descriptive data | 14* | (a) Give characteristics of study participants (eg demographic, clinical, social) and information on exposures and potential confounders | Table 1 and 2 | Table 1 describes patients; Table 2 describes procedure-weighted primary characteristics by treatment. |
|  |  | (b) Indicate number of participants with missing data for each variable of interest | Tables 1 and 2; supplement. | Eight patients lack any assessable recurrence outcome; nine lack an assessable index outcome. |
|  |  | (c) *Cohort study*—Summarise follow-up time (eg, average and total amount) | Section 3.1; Table 1 | Latest-control interval: median 122 days (IQR 78–189) |
| Outcome data | 15* | *Cohort study*—Report numbers of outcome events or summary measures over time | Results; Tables 1, 3 and 4 | TDC-FIT versus TDC: primary recurrence 2/39 versus 35/152; composite failure 6/43 versus 48/165; procedural complications 6/43 versus 12/165. Nine patients died: three in hospital and six after discharge. |
|  |  | *Case-control study—*Report numbers in each exposure category, or summary measures of exposure | *N/A* | *N/A* |
|  |  | *Cross-sectional study—*Report numbers of outcome events or summary measures | *N/A* | *N/A* |
| Main results | 16 | (*a*) Give unadjusted estimates and, if applicable, confounder-adjusted estimates and their precision (eg, 95% confidence interval). Make clear which confounders were adjusted for and why they were included | Section 3.2; Table 3 panels A and B | Primary clustered RR 0.22 (95% CI 0.05–0.93); RD −17.9 percentage points (−28.5 to −7.3); OR 0.18 (0.04–0.84). Adjusted RR 0.16 (0.03–0.74) and OR 0.11 (0.02–0.64). |
|  |  | (*b*) Report category boundaries when continuous variables were categorized | N/A | N/A |
|  |  | (*c*) If relevant, consider translating estimates of relative risk into absolute risk for a meaningful time period | Table 3 panels A and C. |  |

Continued on next page

| Other analyses | 17 | Report other analyses done—eg analyses of subgroups and interactions, and sensitivity analyses | Sections 3.3 and 3.4; Tables 3 and 4. | All patient-level, composite and safety analyses, including nonsignificant findings were reported. |
| --- | --- | --- | --- | --- |
| Discussion | | | | |
| Key results | 18 | Summarise key results with reference to study objectives | Opening Discussion | TDC-FIT was selectively used for higher-risk configurations and was associated with fewer ipsilateral reoperations after eligible procedures. |
| Limitations | 19 | Discuss limitations of the study, taking into account sources of potential bias or imprecision. Discuss both direction and magnitude of any potential bias | Limitations |  |
| Interpretation | 20 | Give a cautious overall interpretation of results considering objectives, limitations, multiplicity of analyses, results from similar studies, and other relevant evidence | Discussion and Conclusion |  |
| Generalisability | 21 | Discuss the generalisability (external validity) of the study results | Discussion | Inferred through the comparison with TDC. |
| Other information | |  | | |
| Funding | 22 | Give the source of funding and the role of the funders for the present study and, if applicable, for the original study on which the present article is based | Funding declaration | No funding |

*Give information separately for cases and controls in case-control studies and, if applicable, for exposed and unexposed groups in cohort and cross-sectional studies.

**Note:** An Explanation and Elaboration article discusses each checklist item and gives methodological background and published examples of transparent reporting. The STROBE checklist is best used in conjunction with this article (freely available on the Web sites of PLoS Medicine at http://www.plosmedicine.org/, Annals of Internal Medicine at http://www.annals.org/, and Epidemiology at http://www.epidem.com/). Information on the STROBE Initiative is available at www.strobe-statement.org.
